# *Limosilactobacillus reuteri* Fermented Brown Rice: A Product with Enhanced Bioactive Compounds and Antioxidant Potential

**DOI:** 10.3390/antiox10071077

**Published:** 2021-07-05

**Authors:** Akanksha Tyagi, Umair Shabbir, Ramachandran Chelliah, Eric Banan-Mwine Daliri, Xiuqin Chen, Deog-Hwan Oh

**Affiliations:** Department of Food Science and Biotechnology, College of Agriculture and Life Sciences, Kangwon National University, Chuncheon 200-701, Korea; akanksha.tyagi001@gmail.com (A.T.); umair336@gmail.com (U.S.); ramachandran865@gmail.com (R.C.); ericdaliri@kangwon.ac.kr (E.B.-M.D.); chenxiuqin0127@kangwon.ac.kr (X.C.)

**Keywords:** brown rice, fermentation, antioxidants, oxidative stress, untargeted metabolomics, UHPLC-QTOF/MS, health benefits

## Abstract

Oxidative stress has been postulated to play a role in several diseases, including cardiovascular diseases, diabetes, and stress-related disorders (anxiety/depression). Presently, natural plant-derived phytochemicals are an important tool in reducing metabolomic disorders or for avoiding the side effects of current medicinal therapies. Brown Rice (*Oryza sativa* L.) is an important part of Asian diets reported as a rich source of bioactive phytonutrients. In our present study, we have analyzed the effect of different lactic acid bacteria (LABs) fermentation on antioxidant properties and in the enhancement of bioactive constituents in Korean brown rice. Therefore, the antioxidant activities and phytochemical analysis were investigated for raw brown rice (BR) and different fermented brown rice (FBR). BR fermented with *Limosilactobacillus reuteri*, showed the highest antioxidant activities among all samples: DPPH (121.19 ± 1.0), ABTS (145.80 ± 0.99), and FRAP (171.89 ± 0.71) mg Trolox equiv./100 g, dry weight (DW). Total phenolic content (108.86 ± 0.63) mg GAE equiv./100 g, DW and total flavonoids content (86.79 ± 0.83) mg catechin equiv./100 g, DW was also observed highest in *Limosilactobacillus reuteri* FBR. Furthermore, phytochemical profiling using ultra-high-performance liquid tandem chromatography quadrupole time-of-flight mass spectrometry (UHPLC-QTOF/MS) and cell antioxidant assay (CAA) revealed *L. reuteri* FBR as a strong antioxidant with an abundance of bioactive compounds such as gamma-aminobutyric acid, coumarin, cinnamic acid, butanoic acid, ascorbic acid, nicotinic acid, and stearic acid. This study expanded current knowledge on the impact of fermentation leading to the enhancement of antioxidant capacity with an abundance of health-related bioactive compounds in BR. The results obtained may provide useful information on functional food production using fermented brown rice.

## 1. Introduction

Oxidative stress is a condition that is caused by an imbalance between antioxidants and free radicals of living organisms. This imbalance occurs due to the excessive production of reactive oxygen species (ROS) or antioxidant deficiency that leads to the damage of aerobic organisms as well as chronic inflammation; referred to as oxidative stress [1]. Lower ROS concentration is important for normal cellular signaling, while excess ROS can cause oxidative damage to DNA, lipids, proteins, and is associated with several chronic diseases [2,3]. The current definition of oxidative stress includes metabolic stress-related pathways that participate in both cellular and extracellular metabolic events. The biology of oxidative stress is extremely complex, with multiple mechanisms at work [4]. Regardless of the mechanism, oxidative stress causes the onset of many diseases including cardiovascular diseases, diabetes, and anxiety or depression which are considered a major public health issue worldwide. As a result, consuming antioxidants to prevent oxidative stress is becoming important for health. Moreover, an increase in the health-consciousness of consumers has increased the demand for nutritional and disease-preventing functional foods, probiotics, prebiotics, and postbiotics. Numerous studies have focused on probiotics, specifically *lactobacilli* strains, that have the potential to act as antioxidants to protect the host from oxidative stress [5]. Some *lactobacilli* strains have been found to quench oxygen free radicals using a chemical antioxidant method. 

Many studies have been reported that phytochemicals (e.g., polyphenols and phenolic acids) derived from natural plants have the potential to target oxidative stress and inflammatory pathways [6,7]. Rice is a staple food (in many countries) that belongs to the grass family (*Oryza sativa*). The total worldwide production of rice was about 769,657,791 tonnes in an area of 167,249,103 ha. Epidemiological studies have shown that the low incidence of chronic diseases in rice-consuming regions can be correlated with rice antioxidants [8,9]. The antioxidant activity and phytochemical content of brown rice (BR) have been recorded in several studies. Components such as as γ-oryzanol, phenolic acids, gamma-aminobutyric acid (GABA), flavonoids, and γ-tocotrienol contribute to the health-promoting properties of brown rice [10].

Evidence supports the effect of solid-state fermentation (SSF) techniques using lactic acid bacteria (LABs) and fungal strains on antioxidant levels and bioactive properties in a variety of substrates, including barley [11], pearl millet [12], and rice [2]. Many researchers, food scientists, and industrialists use the SSF process to enhance the nutritional quality of food and food products. Biological methods are environmentally friendly, relatively safe, and rely on the use of appropriate and specific microorganisms [13]. Our study aimed to provide knowledge to quantify the quality of these phytochemical antioxidants in whole brown rice to meet the needs of food producers and consumers of rice: (1) to analyze the antioxidant properties of differently fermented brown (FBR) rice over raw brown rice (BR); (2) detection of bioactive compounds in raw BR and different LABs fermented brown rice using ultra-high performance liquid chromatography quadrupole time-of-flight mass spectrometry (UHPLC-QTOF/MS), and (3) detection of cellular antioxidant activity of the best LAB fermenting bacterial strain (*L. reuteri* FBR).

## 2. Materials and Methods

### 2.1. Rice Samples

BR (*Oryza sativa L. Var. Japonica*) used in this experiment were obtained from the local market of Chuncheon, Gangwon-do, South Korea. Using an electric mill, raw BR and brown rice after processing (fermentation) were pulverized into a fine powder and sifted through a 40 mesh sieve. Before further extraction, samples were kept at −20 °C.

### 2.2. Chemicals 

Acetonitrile, ethanol, methanol, sodium carbonate, sodium hydroxide, anhydrous sodium acetate, hydrochloric acid, potassium persulfate, acetic acid and sulfuric acid, Dulbecco’s Modified Eagle Medium (DMEM), L-glutamine, 4-(2-hydroxyethyl)piperazine-1-ethane sulfonic acid (HEPES), phosphate-buffered saline (PBS), fetal bovine serum (FBS), penicillin-streptomycin, Hanks’ Balanced Salt Solution (HBSS), and trypsin EDTA were purchased from Daejung chemicals and metals Co., Ltd., South Korea. The phenolic standards and other chemicals such as Folin–Ciocalteu reagent, ABTS (2,2′-Azino-bis (3-ethyl benzothiazoline-6-sulfonic acid), DPPH (2,2-diphenyl-1-picrylhydrazyl); Trolox (6-hydroxy-2,5,7,8-tetramethyl chroman-2-carboxylic acid), p-Coumaric acids, dimethyl sulfoxide (DMSO), TPTZ (2,4,6-Tris (2-pyridyl)-s-triazine), gallic, ferulic, (+)-catechin hydrate (≥98% purity by HPLC), and quercetin dehydrate (≥98% purity by HPLC), 2,2′-azobis (2-amidino propane) dihydrochloride (ABAP); and 2′,7′-dichlorodihydrofluorescein diacetate (DCFH-DA, ≥97% purity), were obtained from Sigma, Seoul, South Korea.

### 2.3. Microorganisms

*Limosilactobacillus reuteri* AKT1 and all other lactic acid bacterial strains used for fermentation in our study were obtained from the Department of Food Science and Biotechnology, Kangwon National University, Korea. LABs were chosen for fermentation in the current study because they demonstrated high GABA (inhibitory neurotransmitter) content and fermentation efficiency in our last study (data not shown) [2]. The bacteria stock culture was kept at −80 °C in MRS broth (Difco), which contained 20% glycerol (*v*/*v*).

### 2.4. Sample Preparation

#### 2.4.1. Brown Rice Fermentation

The LAB’s growth medium consists of sterilized rice powder in distilled water. Before inoculation with lactic acid bacteria, the growth media were autoclaved for 15 min at 121 °C. Different bacterial strains used for fermentation (*L. reuteri* (AKT1), *L. fermentum* (AKT2), and *L. plantarum* (FMP2)) (2 × 10^7^ cfu/mL) were transferred from 12 h (overnight) incubated culture to 100 mL of autoclaved growth media. The media was then incubated for 48 h at 37 °C with 150 rpm agitation, then centrifuged for 10 min at 10,000× *g* and the supernatant was freeze-dried and stored at −20 °C for further study.

#### 2.4.2. Preparation of Extracts

Extraction was done by the method used by Pradeep et al. [14] with some modification. To remove lipids, samples were defatted with hexane using soxhlet equipment. Grounded samples were defatted three times in an orbital shaker at room temperature with hexane (1:5, *w*/*v*, 2 h). Defatted flours were kept at −20 °C till further use. Soluble phenolics from defatted samples (5 g) were extracted in an orbital shaker (RK-2D, DAIHAN scientific, Wonju, Korea) for 1 h at 50 °C with 50% ethanol (1:20 *w*/*v*). The extracts were centrifuged (Union 32R plus, Hanil Science Industrial, Incheon, Korea) at 4000 rpm for 10 min and supernatants were collected and this process was repeated until the third extraction. The supernatants were evaporated at 50 °C and freeze-dried. Before being reconstituted in ethanol, the lyophilized solids were stored at −20 °C. The stock solution of samples was prepared at a concentration of 1 mg/mL that was used throughout the analysis.

### 2.5. Determination of Total Phenolic Content (TPC)

Folin–Ciocalteu colorimetric method was used to measure the TPC reported by Pradeep et al. [15] with few modifications. In brief, 100 μL of the sample extract or standard (gallic acid solution) or 95% (*v*/*v*) methanol as blank was treated with 200 μL of Folin–Ciocalteu reagent for a short duration of 6 min. The mixture was then alkalized with 1 mL of Na_2_CO_3_ 700 mM. After being kept in dark conditions for 90 min, the SpectraMax i3 plate reader (Molecular Devices Korea, LLC, Seoul, Korea) was used to measure the absorbance at 760 nm wavelength. Gallic acid was used as a standard to calculate the TPC and results were expressed as milligrams of gallic acid equivalent per 100 g of sample (mg GAE equiv./100 g, DW).

### 2.6. Determination of Total Flavonoid Content (TFC)

TFC was analyzed using the method of Pradeep et al. [15] with few modifications. Briefly, a 200 μL sample extract was combined with 75 μL of NaNO_2_ (50 gL^−1^) followed by the addition of 1 mL of distilled water. Then, the reaction mixture was allowed to settle for 5 min and then 75 μL of AlCl_3_ (100 gL^−1^) was added. After 6 min, 600 μL of distilled water followed by 500 μL of 1 M NaOH were added. The SpectraMax i3 plate reader (Molecular Devices Korea, LLC) was used to measure the absorbance at 510 nm wavelength. Catechin was used as a standard and results were expressed as milligram catechin equivalents per 100 g of sample (mg catechin equiv./100 g, DW).

### 2.7. Determination of Antioxidant Activities of BR

#### 2.7.1. DPPH Radical Scavenging Activity

DPPH activity was determined by the methods of Chang et al. [16] after slight modifications. In short, 100 μL of the sample extract or standard (Trolox) or blank (methanol) was mixed with freshly prepared 100 μL of 500 μM DPPH solution (dissolved in methanol) in a 24-well microplate and incubated at room temperature for 30 min. The absorbance was measured at 515 nm wavelength. The Trolox concentration plot with DPPH radical scavenging activity was used as a baseline curve. DPPH values were expressed as mg Trolox equivalent per 100 g of sample (mg Trolox equiv./100 g, DW) using the following formula:

DPPH Radical Scavenging Activity (%) = (A_c_ − A_e_)/A_b_ × 100

where A_e_ represents the absorbance of the extract or standard and A_c_ represents the absorbance value of the blank sample.

#### 2.7.2. ABTS Radical Scavenging Activity

ABTS assay was carried out as described by Chang et al. [16] with little modifications. ABTS stock solution was prepared by mixing 2.45 mmol/L of potassium persulfate with 7 mmol/L of ABTS solution (1:1, *v*/*v*) and kept in the dark for 12–16 h at room temperature. The ABTS + reagent was constantly diluted with methanol until 0.700 ± 0.020 absorbance at 734 nm wavelength. Afterwards, 100 μL of extracts or standards were mixed with ABTS + solution (1 mL) and absorbance was measured at 734 nm. The per cent inhibition of ABTS was measured using the same formula as for the DPPH assay (mentioned above). ABTS values were expressed as mg Trolox equiv./100 g, DW using the Trolox standard curve.

#### 2.7.3. Ferric Reducing Antioxidant Power (FRAP)

FRAP assay was analyzed using the method as documented by Xiang et al. [17] with little modifications. In short, 0.1 mL of extracts were combined with a FRAP reagent of 3.9 mL that was prepared using acetate buffer (50 mL, 0.3 M, pH 3.6), Tripyridyl Triazine (5 mL, TPTZ) solution (10 mM of TPTZ in 40 mM of HCl) and FeCl_3_ · 6H_2_O (5 mL, 20 mM) and kept for 10 min at 37 °C, then absorbance was taken at 593 nm wavelength. These findings were expressed as mg Trolox equiv./100 g, DW.

### 2.8. Identification of BR Bioactive Compounds Using UHPLC-Q-TOF-MS/MS 

The bioactive compounds of BR samples were analyzed using UHPLC Q-TOF-MS/MS (SCIEX Exion LC AD system, Framingham, MA, USA) according to the protocol previously conducted in our laboratory by Tyagi et al., and Daliri et al. [2,18]. The mass spectrometric analysis was conducted in both positive (ESI+) and negative (ESI−) ion modes. In summary, UHPLC Q-TOF-MS/MS system was fitted with different components including an autosampler, photodiode array detector, and controller. The analytical column used in the analysis was 100 mm × 3 mm Accucore C18 column (Thermo Fisher Scientific, Waltham, MA, USA). Later, the sample (10 µL) was injected by autosampler and eluted into the column with a binary mobile phase consisting of solvent A (water with 0.1% of formic acid) and solvent B (methanol). A flow rate (0.4 mL/min) with a linear gradient programmed for 25 min was used in this analysis. Under these conditions, the scanning time was approximately 1 s. The bioactive compounds of BR were identified by using a metabolomics workbench.

### 2.9. Cell Viability Assay 

After performing antioxidant assays and untargeted metabolomics of raw BR and different LABs treated BR samples, the best (*L. reuteri* FBR) sample was selected for further cell line analysis. To test the viability in Caco-2 cell lines, the *L. reuteri* FBR sample was analyzed using a laboratory EZ-cytox assay. WST of EZ-cytox exists in the respiratory chain of mitochondria and is active only in living cells. Briefly, Caco-2 cells in the growth medium were seeded on a 96-well plate at a density of 4 × 10^4^ cells/well. The growth medium was removed and the cells were washed using PBS after 24 h of incubation at 37 °C with 5 per cent CO_2_. Then, 100 μL of growth medium with various sample extract concentrations was applied. In the control group, a medium without sample extract was added. After 12 h of incubation at 37 °C with 5 per cent CO_2_, 10 μL WST-1 solution was added to each sample. The 96-well plate was left for 10 min at 37 °C and the absorbance was measured at 455 nm by SpectraMax i3 plate reader. If a concentration of sample extract reduced the cell viability by >10%, then the extract at this concentration was cytotoxic.

$$\mathrm{cell}\;\mathrm{viability}\;(\%)\;=\;\frac{\mathrm{mean}\;\mathrm{absorbance}\;\mathrm{in}\;\mathrm{test}\;\mathrm{well}}{\mathrm{mean}\;\mathrm{absorbance}\;\mathrm{in}\;\mathrm{control}\;\mathrm{well}}\;\times \;100$$

### 2.10. Cellular Antioxidant Activity (CAA) 

The formation of ROS within cells was investigated using oxidation sensitive DCFH-DA probes by the method of Ti et al. [19] with slight modifications. In brief, Caco-2 cells were grown overnight at a density of 6 × 10^4^ cells per well in black 96-well microplates. Later, PBS (50 μL) was used to wash the cells after 2 h of pretreatment with various concentrations of sample extracts (0.5–5 mg/mL) and 100 μL of DCFH-DA (25 μmol/L). Then, 100 μL of DMEM medium (composed of 600 µmol L^−1^ ABAP) was inoculated in each well excluding the blank well, which received 100 μL of DMEM medium without ABAP. The fluorescence was measured using a plate reader SpectraMax i3 plate reader (Seoul, South Korea) and wavelengths of 485 nm excitation and 538 nm emission for 13 cycles (5 min each). The formula used for calculating the CAA unit was as follows:

CAA unit = % reduction = (1 − (∫ SA/∫CA)) × 100

where ∫SA and ∫CA denote the integral areas under the sample and control time-fluorescence value curves, respectively.

### 2.11. Statistical Analysis

GraphPad Prisma 8.0 was used to analyze the data. Using the SPSS program and GraphPad Prism 8.0, differences in mean values between brown rice samples of phenolic extracts were calculated using one-way variance analysis (ANOVA) followed by a Tukey test at *p* < 0.05 significance stage. The findings were referred to as mean standard deviation (SD).

The empirical formula was used to identify compounds such as PubChem (https://pubchem.ncbi.nlm.nih.gov/ accessed on 10 June 2021) or ChemSpider (http://www.chemspider.com/ accessed on 10 June 2021). ClustVis program (http://biit.cs.ut.ee/clustvis/ accessed on 10 June 2021), XCMS online (Metlin) (https://xcmsonline.scripps.edu accessed on 10 June 2021), Paleontological Statistics (PAST), and clustVis (https://biit.cs.ut.ee/clustvis/ accessed on 10 June 2021) were used in multivariate statistical analyses, including heat maps [20].

## 3. Results and Discussion

### 3.1. TPC and TFC

The TPC and TFC of all four samples are shown in (Table 1) as mean ± SD of triplicate analyses with statistically significant differences (Tukey and Duncan test *p* ≤ 0.05). TPC ranged between 16.08 ± 0.49 to 108.86 ± 0.63 mg GAE equiv./100 g, DW. TPC content was found lowest in raw BR samples 16.08 ± 0.49 and highest in *L. reuteri* FBR 108.86 ± 0.63. Hydrolysis by enzymes during fermentation usually increases total phenolic content, as observed in this study. TPC increases with fermentation, *L. fermentum* FBR (75.00 ± 0.017 mg GAE/100 g, DW), *L. plantarum* FBR (96.87 ± 0.94 mg GAE equiv./100 g, DW), and *L. reuteri* FBR (108.86 ± 0.63 mg GAE equiv./100 g, DW), compared with raw BR. This study shows higher TPC content of BR than reported by [21,22].

TFC was found higher in *L. reuteri* FBR 86.79 ± 0.83 mg catechin equiv./100 g, DW, followed by *L. plantarum* FBR and *L. fermentum* FBR (66.28 ± 0.71 and 54.77 ± 1.02 mg catechin equiv./100 g, DW) (Table 1). TFC content was lower than TPC in BR samples. TFC in our study was higher than previously reported by Huang et al. [23], similar to TPC, but both TPC and TFC were found lower than reported by Gong et al. [24]. These differences in BR values of different researchers could be because of genotype, cultivation landscape, and climate conditions. In addition, it is worth noting that the phenolic content can be significantly influenced by different extraction solvents and procedures.

### 3.2. Antioxidant Assay (DPPH, ABTS, FRAP)

Various methodologies including reducing capacity, free radical scavenging, lipid peroxidation inhibition, and metal ion chelation have been studied to explain how rice extracts have shown effective antioxidant potential [25]. In recent years, the fermentation process is thought to be an effective method for increasing antioxidant activity in cereals. The antioxidant activity of raw and different LABs fermented brown rice samples were assessed using the DPPH, ABTS, and FRAP assays in the current study. The antioxidant values of DPPH, ABTS, and FRAP of phenolic extracts of BR and differently treated BR samples were presented in Table 1, respectively.

The detection of scavenging activity of the 2,2′-diphenyl-1-picrylhydrazyl (DPPH) radical by spectrophotometry is one of the most commonly used assays in the determination of antioxidant activity in natural products. The DPPH radical scavenging activity was found highest in the *L. reuteri* FBR (121.19 ± 1.0 mg Trolox equiv./100 g, DW), followed by *L. plantarum* and *L. fermentum* FBR (95.03 ± 0.81, 90.93 ± 0.74 mg Trolox equiv./100 g, DW). The lowest values were observed in raw BR (17.342 ± 0.44 mg Trolox equiv./100 g, DW). 

Similarly, ABTS is considered an important method for determining radical scavenging activity in grains and plant materials. Furthermore, FRAP assay was originally designed to assess plasma antioxidant ability but has also been commonly used in a wide variety of pure compounds and biological samples to determine antioxidant capacity. It measures absorption changes caused by the formation of blue iron (II) from colorless iron oxide (III). In the present research, the same trend as DPPH was observed in ABTS and FRAP assays. ABTS activity was measured highest in *L. reuteri* FBR (145.80 ± 0.99 mg Trolox equiv./100 g, DW) (Table 1). In ABTS, the lowest activity was also by raw BR. Similarly, FRAP was found highest in FBR (171.89 ± 0.71 mg Trolox equiv./100 g, DW) followed by *L. plantarum* and *L. fermentum* FBR. As a result of different antioxidants assays, *L. reuteri* FBR showed the highest activity among all samples. These findings were higher than earlier reports by Lin et al. [26] and IIowefah et al. [21] in fermented BR. Furthermore, other studies have reported the high antioxidant activity of BR [27].

### 3.3. Untargeted Metabolomics Using UHPLC Q-TOF-MS/MS in Brown Rice Samples

UHPLC Q-TOF-MS/MS detection is considered a gold standard technique for the precise detection and quantification of a wide variety of components. Therefore, in this study, we have used this detection technique for the identification of phenolic compounds in brown rice. 

#### 3.3.1. Phenolic Compounds

**Figure 1:**
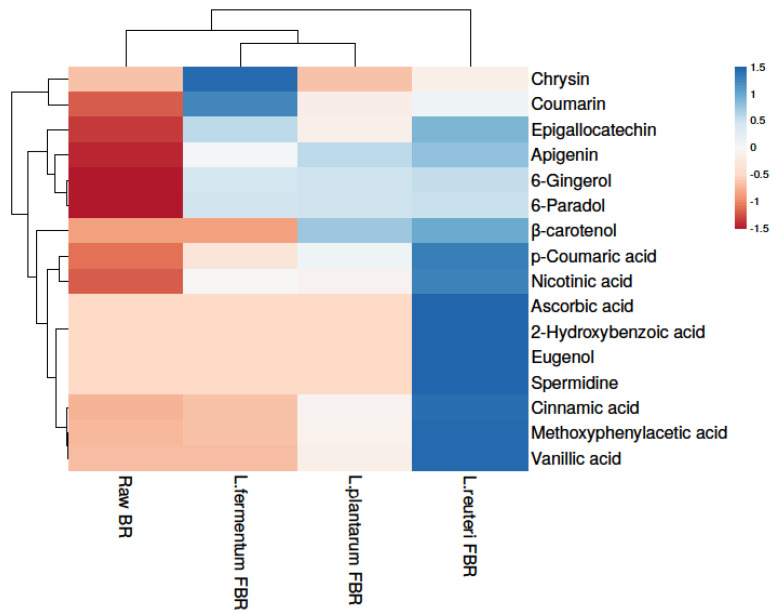
Heat map showing levels of phenolic compounds in raw and LABs fermented BR samples.

In the present research, the phenolic compositions of BR treated with different fermentation bacteria were selected and positively or tentatively identified by UHPLCQ-TOF-MS/MS. Phenolic identification and characterization were achieved by comparing our results with mass spectral literature evidence and cross-referencing it with other available spectral databases, such as Metlin and Metabolomics Workbench. A total of 15 phenolic compounds were tentatively found from our soluble extracts of raw BR, *L. reuteri* FBR, *L. fermentum* FBR, and *L. plantarum* FBR respectively, as shown in Table 2. In the ethanol extract, we identified compounds 1 to 14 at different adduct charges [M − H]^−^ and [M − H]^+^ which are identified by comparing with mass spectral libraries, XCMS online (Metlin), and Metabolomics Workbench. Heat map analysis was used for clustering phenolic compounds based on their concentrations (Figure 1) where the color scheme from blue to red shows concentration in decreasing order.

Results showed that the highest phenolic compounds were detected in the *L. reuteri* FBR sample. Because phenolic compounds are not readily available, they typically occur in cereals in esterified linkages to the cereal wall matrix [28]. Fermentation is considered to be a possible strategy to release insoluble or bound phenolic compounds and thus leading to improve the poor bioavailability of grain phenolics. Comparing different fermenting bacteria in the present study we found that *L. reuteri* fermentation releases most of the phenolic compounds compared with other bacterial strains and thus improves the bioavailability and bioaccessibility of cereal grains such as brown rice phenolics [29]. Many phenolic compounds detected in the current study such as p-coumaric acid [30], ascorbic acid [31], cinnamic acid [32], and vanillic acid [33] are already reported in the literature for their strong antioxidant capacities.

#### 3.3.2. Levels of Amino Acid in Brown Rice

**Figure 2:**
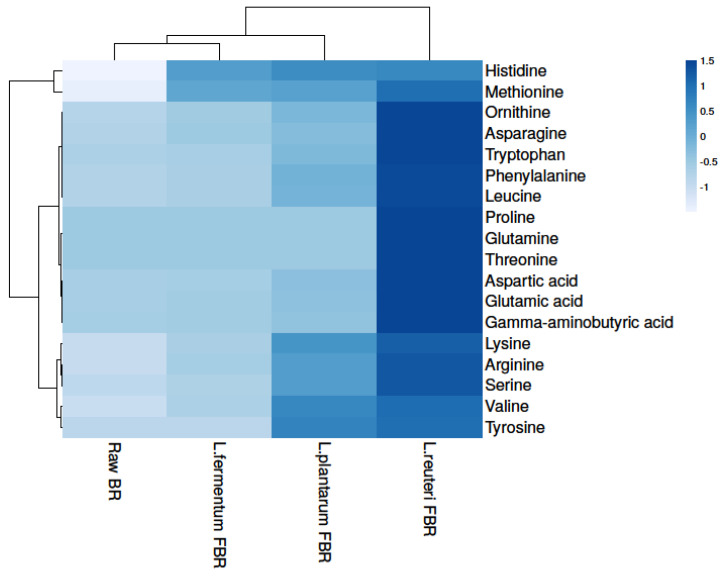
Heat map showing levels of amino acids in raw and different fermented BR samples.

In the growth and development of organisms, amino acids play an important role and can also improve the taste of food. In our present study, a total of 18 amino acids were detected in raw and differently fermented BR samples (Figure 2 and Table 3) which shows statistically significant differences from each other after comparing their levels. Raw BR contained the least number of amino acids, which may be due to more bound molecules with the parent, whereas fermentation leads to an increase in amino acid content. The levels of amino acids were detected highest in the *L. reuteri* FBR sample which might strain-specific as fermentation microorganisms produce enzymes that lead to the formation of several metabolites and bioactive compounds from the food matrix [34]. In the ethanol extract, we found levels of some essential amino acids (tryptophan, lysine, methionine, and histidine), as well as certain conditionally essential amino acids (arginine, ornithine, serine, and glutamine), increased drastically after fermentation (Figure 2 and Table 3). The identification was done by comparing with mass spectral libraries, XCMS online (Metlin) and Metabolomics Workbench. In amino acids, *L. reuteri* FBR also shows the highest number of amino acids content as observed in phenolic compounds. 

#### 3.3.3. Level of Fatty Acid in Brown Rice Samples

**Figure 3:**
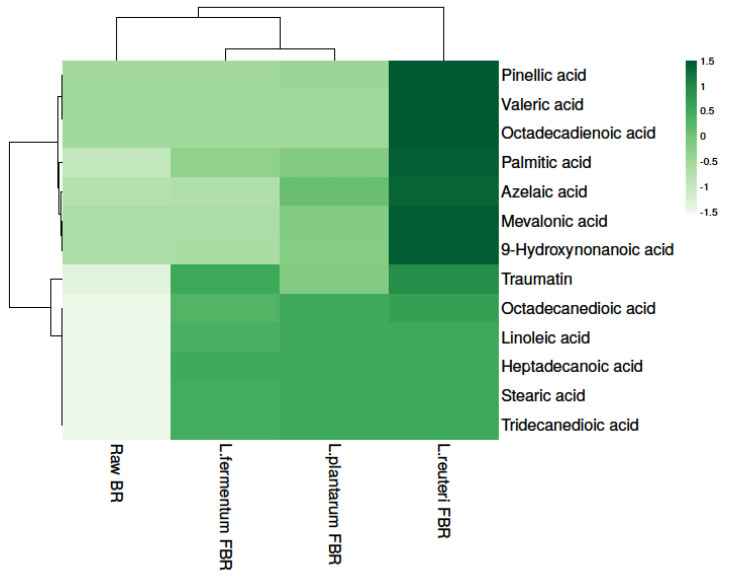
Heat map showing levels of fatty acids in raw and different LABs fermented BR samples.

In particular, fermentation has been proposed as a tool for enhancing foods’ nutritional values, both in terms of enhanced bioavailability of bioactive components as well as the production of health-promoting end-products. Due to their proven benefit, in the last decade, short-chain fatty acids (SCFAs) have emerged as some of the most researched compounds. In the present study, 13 fatty acids were detected in raw and different LABs fermented BR samples (Table 4) and fatty acid levels were found to be significantly different in all samples. The results show that the highest levels of fatty acids were found in *L. reuteri* FBR. Heat map analysis was used for separating fatty acids based on the different concentrations, represented in different shades of green (dark to light) in decreasing order (Figure 3).

### 3.4. Cell Viability Assay and Cellular Antioxidant Activity (CAA) 

#### 3.4.1. Cell Viability Assay

**Figure 4:**
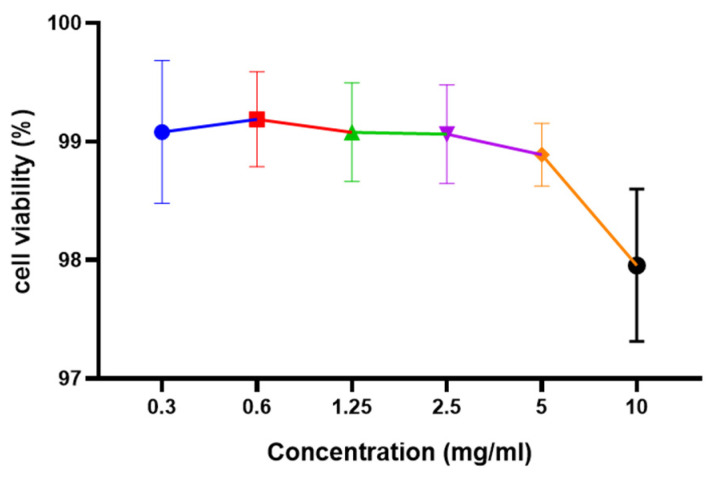
Effect of *L. reuteri* FBR extracts on viability of Caco-2 cells analyzed by Ez cytox assay kit. Cells were treated with an increased concentration of *L. reuteri* FBR extracts for 12 h. Data are represented as means ± standard deviations (*n* = 3).

Cytotoxicity is regarded as an important step in determining the suitability and further applications of any food extract. Using the Ez cytox assay kit, the cytotoxic effect of *L. reuteri* FBR extracts at 0.3–10 mg/mL concentrations was investigated using Caco-2 cell lines. Figure 4 depicts the cell viability results of the extract after 12 h of incubation. It was observed that cell viability was not much decreased after increasing the concentration up to 10 mg/mL. No significant differences were observed in cytotoxicity assay by using 0.3–10 mg/mL concentrations (Figure 4). The extract was observed to be non-toxic after 12 h assay as extract still shows about 97 per cent of cell viability. Our results were found similar to the results presented by Yue et al. [35].

#### 3.4.2. Cellular Antioxidant Activity (CAA)

**Figure 5:**
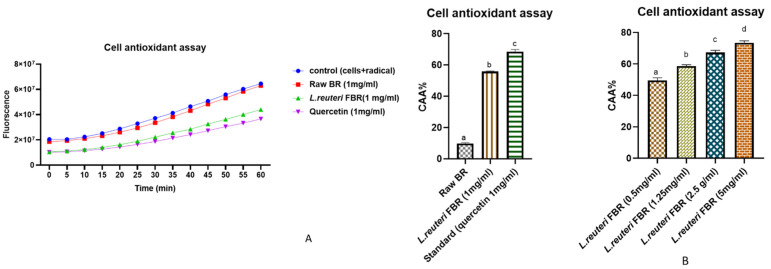
In Caco-2 cells, peroxyl radical-induced oxidation of DCFH to DCF and ROS inhibition by raw BR and *L. reuteri* FBR extract (**A**,**B**) showing the effect of dose-dependent inhibition of *L. reuteri* FBR extracts (0.5–5 mg/mL). Data were represented as means ± standard deviations (*n* = 3) with one way ANOVA. The columns with different letters (a–d) show significant differences using Tukey’s test at *p* < 0.05.

The effect of pretreatment of Caco-2 cells with *L. reuteri* fermented extract of brown rice on intracellular reactive oxygen species (ROS) was determined using a cell-based assay. The fluorescent probe DCFH-DA is used as an indicator of ROS and oxidative stress in our study. The nonionic and nonpolar DCFH-DA probe diffuses passively into cells before being hydrolyzed by intracellular esterases to form nonfluorescent 2′,7′-dichlorofluorescein (DCFH). Later in the presence of ROS, DCFH that is trapped inside cells is oxidized into fluorescent 2′,7′-dichlorofluorescein (DCF) [36]. When the cellular antioxidant defense system fails to compensate for ROS production, oxidative stress occurs. This reaction can be slowed down using bioactive compounds, preventing the generation of DCF. Following the uptake of antioxidant compounds can be accomplished on the cell membrane surface or within the cell [37]. We evaluated the effect of our *L. reuteri* FBR extract against oxidative stress in Caco-2 cells. In our study, ABAP was chosen as an intracellular oxidizing agent to simulate oxidative stress in cells. 600 µmol L^−1^ ABAP was chosen as the optimal concentration to induce oxidation. As represented in Figure 5A, CAA values in *L. reuteri* FBR extract were observed to be 5.7 times higher than the raw BR sample at a concentration of 1mg/mL. Our results indicate that extracts reduced ROS levels at rest in a dose-dependent manner (Figure 5B); CAA values were increased with concentration (0.5 mg/mL to 5 mg/mL) from 49.50 ± 1.67% to 72.49 ± 1.23%. The strength of inhibition strongly followed a curvilinear pattern as *L. reuteri* FBR extract concentrations increased. A similar effect was previously observed in the study of Grauzdytė et al., where they observed *Phyllanthus phillyreifolius* extracts in HEK-293 cells [38], and the study of Kellett et al. [39] in Caco-2 cells.

## 4. Conclusions

In our study, we discovered that *L. reuteri* FBR had higher antioxidant activity as well as a higher concentration of phenolics and flavonoids among all LABs used for the study. This shows the ability of *L. reuteri* as a promising fermentation strain to increase the bioavailability of cereals or grains in producing health-promoting functional materials. *L. reuteri* fermentation improves phenolic constituents and antioxidant activity of BR, improves food quality, and confers organoleptic characteristics. Furthermore, we discovered that *L. reuteri* FBR enhanced the production of essential amino acids and fatty acids using untargeted metabolomics. The present study has provided information on bioactive compounds and antioxidant activities as well as the cellular antioxidant capacities of *L. reuteri* FBR. These data are required for the processing of the whole BR and its products for the pharmaceutical and food markets. As a result, new strategies and collaborations among industry, researchers, and relevant agencies are required to publicize whole grain consumption. Additionally, the current research is part of ongoing efforts to increase the added value of brown rice production and use in the prevention of human chronic diseases caused by oxidative stress. Moreover, these findings also make this sample a promising material for the development of health-promoting functional food. Whereas, it is necessary to perform more research into the mechanisms of different types of fermentation (solid and liquid-state) on single/pure phenolic compounds and antioxidant properties. Furthermore, in vivo models should be used to study the bioavailability and absorption of phenolic compounds in the gut.

## Figures and Tables

**Table 1:** Total antioxidants DPPH, ABTS, FRAP, total phenolic content (TPC), and total flavonoid content (TFC) of raw and different LABs fermented BR samples.

S.NO	Sample	DPPH (mg Trolox Equiv./100 g, DW)	ABTS (mg Trolox Equiv./100 g, DW)	FRAP (mg Trolox Equiv./100 g, DW)	TPC (mg Gallic Acid Equiv./100 g, DW)	TFC (mg Catechin Equiv./100 g, DW)
1	Raw BR	17.342 ± 0.44 ^d^	19.86 ± 0.86 ^d^	18.01 ± 0.74 ^d^	16.08 ± 0.49 ^d^	13.42 ± 0.80 ^d^
2	*L. fermentum* FBR	90.93 ± 0.74 ^c^	92.10 ± 0.81 ^c^	99.59 ± 1.60 ^b^	75.00 ± 0.01 ^c^	54.77 ± 1.02 ^c^
3	*L. reuteri* FBR	121.19 ± 1.0 ^a^	145.80 ± 0.99 ^a^	171.89 ± 0.71 ^a^	108.86 ± 0.63 ^a^	86.79 ± 0.83 ^a^
4	*L. plantarum* FBR	95.03 ± 0.81 ^b^	97.73 ± 0.47 ^b^	104.31 ± 0.48 ^b^	96.87 ± 0.94 ^b^	66.28 ± 0.71 ^b^

BR—Brown rice, FBR—fermented brown rice. Results were expressed as mean ± SD of triplicate analyses. Different alphabetical letters in each column represent statistically significant differences (Tukey and Duncan test *p* ≤ 0.05) DW, dry weight sample.

**Table 2:** Phenolic compounds detected in raw and LABs fermented BR.

S. No	Sample Name	Retention Time (min)	Peak Area	Adduct/Charge	Precursor Mass	Found at Mass	Molecular Formula	Tentative Phenolic Compound
1	Raw BR	Nd	Nd	[M − H]−	Nd	Nd	C_25_H_36_O	Beta-carotenol
	*L. plantarum* FBR	45.56	4.53 × 10^5^	[M − H]−	353.268	353.187		
	*L. fermentum* FBR	Nd	Nd	[M − H]−	Nd	Nd		
	*L. reuteri* FBR	45.45	5.19 × 10^5^	[M − H]−	353.268	353.284		
2	Raw BR	Nd	Nd	[M + H]+	Nd	Nd	C_10_H_12_O_2_	Eugenol
	*L. plantarum* FBR	Nd	Nd	[M + H]+	Nd	Nd		
	*L. fermentum* FBR	Nd	Nd	[M + H]+	Nd	Nd		
	*L. reuteri* FBR	20.81	2.24 × 10^5^	[M + H]+	179.107	179.1067		
3	Raw BR	33.80	2.20 × 10^5^	[M − H]−	293.177	293.1761	C_17_H_26_O_4_	6-Gingerol
	*L. plantarum* FBR	33.80	2.08 × 10^6^	[M − H]−	293.177	293.176		
	*L. fermentum* FBR	33.81	2.03 × 10^6^	[M − H]−	293.177	293.1761		
	*L. reuteri* FBR	33.80	2.13 × 10^6^	[M − H]−	293.177	293.1762		
4	Raw BR	Nd	Nd	[M + H]+	Nd	Nd	C_15_H_10_O_4_	Chrysin
	*L. plantarum* FBR	Nd	Nd	[M + H]+	Nd	Nd		
	*L. fermentum* FBR	14.78	4.09 × 10^5^	[M + H]+	253.052	253.0524		
	*L. reuteri* FBR	14.81	1.01 × 10^5^	[M + H]+	253.052	253.0527		
5	Raw BR	Nd	Nd	[M + H]+	Nd	Nd	C_16_H_8_N_2_O_5_	Apigenin
	*L. plantarum* FBR	14.79	4.68 × 10^5^	[M + H]+	269.047	269.0457		
	*L. fermentum* FBR	14.78	3.38 × 10^5^	[M + H]+	269.047	269.0457		
	*L. reuteri* FBR	14.78	5.15 × 10^5^	[M + H]+	269.047	269.0458		
6	Raw BR	Nd	Nd	[M + H]+	Nd	Nd	C_9_H_6_O_2_	Coumarin
	*L. plantarum* FBR	1.92	1.24 × 10^5^	[M + H]+	147.044	147.0444		
	*L. fermentum* FBR	1.87	2.94 × 10^5^	[M + H]+	147.044	147.0447		
	*L. reuteri* FBR	1.90	1.55 × 10^5^	[M + H]+	147.044	147.0445		
7	Raw BR	Nd	Nd	[M + H]+	Nd	Nd	C_15_H_14_O_7_	Epigallocatechin
	*L. plantarum* FBR	12.26	7.44 × 10^5^	[M + H]+	305.071	305.067		
	*L. fermentum* FBR	12.28	1.20 × 10^6^	[M + H]+	305.071	305.067		
	*L. reuteri* FBR	12.28	1.38 × 10^6^	[M + H]+	305.071	305.067		
8	Raw BR	Nd	Nd	[M + H]+	Nd	Nd	C_7_H_19_N_3_	Spermidine
	*L. plantarum* FBR	Nd	Nd	[M + H]+	Nd	Nd		
	*L. fermentum* FBR	Nd	Nd	[M + H]+	Nd	Nd		
	*L. reuteri* FBR	0.96	8.75 × 10^5^	[M + H]+	188.176	188.1761		
9	Raw BR	38.06	3.21 × 10^4^	[M − H]−	277.182	277.1812	C_17_H_26_O_3_	6-Paradol
	*L. plantarum* FBR	38.06	4.38 × 10^5^	[M − H]−	277.182	277.1812		
	*L. fermentum* FBR	38.08	4.33 × 10^5^	[M − H]−	277.182	277.1813		
	*L. reuteri* FBR	38.06	4.42 × 10^5^	[M − H]−	277.182	277.1812		
10	Raw BR	Nd	Nd	[M − H]−	Nd	Nd	C_9_H_8_O_2_	Cinnamic acid
	*L. plantarum* FBR	4.01	3.26 × 10^5^	[M − H]−	147.046	147.0455		
	*L. fermentum* FBR	4.03	4.46 × 10^4^	[M − H]−	147.046	147.0456		
	*L. reuteri* FBR	3.98	1.09 × 10^6^	[M − H]−	147.046	147.0454		
11	Raw BR	Nd	Nd	[M + NH4]+	Nd	Nd	C_9_H_8_O_3_	p-Coumaric acid
	*L. plantarum* FBR	1.86	1.06 × 10^6^	[M + NH4]+	182.081	182.0813		
	*L. fermentum* FBR	1.92	6.90 × 10^5^	[M + NH4]+	182.081	182.0813		
	*L. reuteri* FBR	1.87	2.13 × 10^6^	[M + NH4]+	182.081	182.0812		
12	Raw BR	Nd	Nd	[M − H]−	Nd	Nd	C_9_H_10_O_3_	Methoxyphenylacetic acid
	*L. plantarum* FBR	15.28	5.20 × 10^6^	[M − H]−	165.057	165.0558		
	*L. fermentum* FBR	15.29	4.09 × 10^5^	[M − H]−	165.057	165.056		
	*L. reuteri* FBR	15.27	1.92 × 10^7^	[M − H]−	165.057	165.0557		
13	Raw BR	Nd	Nd	[M − H]−	Nd	Nd	C_7_H_6_O_3_	Sesamol/2-Hydroxybenzoic acid
	*L. plantarum* FBR	Nd	Nd	[M − H]−	Nd	Nd		
	*L. fermentum* FBR	Nd	Nd	[M − H]−	Nd	Nd		
	*L. reuteri* FBR	19.63	3.24 × 10^5^	[M − H]−	137.025	137.0249		
14	Raw BR	Nd	Nd	[M − H]−	Nd	Nd	C_8_H_8_O	Vanillic acid
	*L. plantarum* FBR	15.28	3.18 × 10^5^	[M − H]−	119.051	119.0504		
	*L. fermentum* FBR	Nd	Nd	[M − H]−	Nd	Nd		
	*L. reuteri* FBR	15.27	1.27 × 10^6^	[M − H]−	119.051	119.0504		
15	Raw BR	Nd	Nd	Nd	Nd	Nd	C_6_H_8_O_6_	Ascorbic acid (Vitamin C)
	*L. plantarum* FBR	Nd	Nd	Nd	Nd	Nd		
	*L. fermentum* FBR	Nd	Nd	Nd	Nd	Nd		
	*L. reuteri* FBR	1.02	3.05 × 10^3^	[M + H]+	209.009	209.0107		

Nd—not detected, BR—brown rice, and FBR—fermented brown rice.

**Table 3:** Amino acids detected in raw and LABs fermented brown rice.

S. No	Sample Name	Retention Time (Min)		Peak Area	Adduct/ Charge	Precursor Mass	Found at Mass	Formula Finder Result	Amino Acid
1	Raw BR	1.00		1.52 × 10^3^	[M + H]+	156.077	156.077	C_6_H_9_N_3_O_2_	Histidine
	*L. plantarum* FBR	1.00		7.31 × 10^5^	[M + H]+	156.077	156.077		
	*L. fermentum* FBR	1.02		6.33 × 10^5^	[M + H]+	156.077	156.0771		
	*L. reuteri* FBR	1.02		7.45 × 10^5^	[M + H]+	156.077	156.0771		
2	Raw BR	ND		ND	[M − H]−	ND	ND	C_6_H_14_N_2_O_2_	Lysine
	*L. plantarum* FBR	1.02		9.16 × 10^5^	[M − H]−	145.099	145.0982		
	*L. fermentum* FBR	1.02		2.17 × 10^5^	[M − H]−	145.099	145.0984		
	*L. reuteri* FBR	1.02		1.40 × 10^6^	[M − H]−	145.099	145.0983		
3	Raw BR	ND		ND	[M + H]+	ND	ND	C_5_H_11_NO_3_S	Methionine
	*L. plantarum* FBR	1.17		5.66 × 10^5^	[M + H]+	166.053	166.0536		
	*L. fermentum* FBR	1.17		5.33 × 10^5^	[M + H]+	166.053	166.0537		
	*L. reuteri* FBR	1.18		8.41 × 10^5^	[M + H]+	166.053	166.0536		
4	Raw BR	1.17		2.56 × 10^2^	[M − H]−	146.047	146.0457	C_5_H_9_NO_4_	Glutamic acid
	*L. plantarum* FBR	1.47		3.18 × 10^5^	[M − H]−	146.047	146.046		
	*L. fermentum* FBR	1.47		4.58 × 10^4^	[M − H]−	146.047	146.046		
	*L. reuteri* FBR	1.47		2.65 × 10^6^	[M − H]−	146.047	146.0458		
5	Raw BR	ND		ND	[M + H]−	ND	ND	C_4_H_9_NO_2_	Gamma-aminobutyric acid
	*L. plantarum* FBR	1.16		1.38 × 10^5^	[M − H]−	102.057	102.056		
	*L. fermentum* FBR	1.16		2.76 × 10^4^	[M − H]−	102.057	102.0563		
	*L. reuteri* FBR	1.16		1.28 × 10^6^	[M − H]−	102.057	102.0561		
6	Raw BR	ND		ND	[M + H]+	ND	ND	C_6_H_14_N_4_O_2_	Arginine
	*L. plantarum* FBR	1.11		2.81 × 10^6^	[M + H]+	175.118	175.1183		
	*L. fermentum* FBR	1.14		9.09 × 10^5^	[M + H]+	175.118	175.1194		
	*L. reuteri* FBR	1.11		4.99 × 10^6^	[M + H]+	175.118	175.1184		
7	Raw BR	ND		ND	[M − H]−	ND	ND	C_5_H_11_NO_2_	Valine
	*L. plantarum* FBR	1.49		6.15 × 10^5^	[M − H]−	116.073	116.0717		
	*L. fermentum* FBR	1.50		1.37 × 10^5^	[M − H]−	116.073	116.0718		
	*L. reuteri* FBR	1.45		7.61 × 10^5^	[M − H]−	116.073	116.0719		
8	Raw BR	1.14		1.19 × 10^3^	[M − H]−	132.031	132.0307	C_4_H_7_NO_4_	Aspartic acid
	*L. plantarum* FBR	1.15		7.26 × 10^4^	[M − H]−	132.031	132.0303		
	*L. fermentum* FBR	1.15		8.76 × 10^3^	[M − H]−	132.031	132.0305		
	*L. reuteri* FBR	1.12		4.89 × 10^5^	[M − H]−	132.031	132.0302		
9	Raw BR	ND		ND	[M − H]−	ND	ND	C_9_H_11_NO_2_	Phenylalanine
	*L. plantarum* FBR	4.01		2.26 × 10^6^	[M − H]−	164.072	164.0718		
	*L. fermentum* FBR	4.03		3.50 × 10^5^	[M − H]−	164.072	164.072		
	*L. reuteri* FBR	3.98		7.21 × 10^6^	[M − H]−	164.072	164.0718		
10	Raw BR	ND		ND	[M − H]−	ND	ND	C_5_H_12_N_2_O_2_	Ornithine
	*L. plantarum* FBR	1.01		1.53 × 10^5^	[M − H]−	131.084	131.0827		
	*L. fermentum* FBR	1.01		6.67 × 10^4^	[M − H]−	131.084	131.0828		
	*L. reuteri* FBR	1.01		5.35 × 10^5^	[M − H]−	131.084	131.0827		
11	Raw BR	1.12		3.31 × 10^2^	[M − H]−	104.036	104.0353	C_3_H_7_NO_3_	Serine
	*L. plantarum* FBR	1.12		3.06 × 10^5^	[M − H]−	104.036	104.0353		
	*L. fermentum* FBR	1.13		4.70 × 10^4^	[M − H]−	104.036	104.0356		
	*L. reuteri* FBR	1.12		5.73 × 10^5^	[M − H]−	104.036	104.0353		
12	Raw BR	ND		ND	[M − H]−	ND	ND	C_6_H_13_NO_2_	Leucine
	*L. plantarum* FBR	2.46		1.95 × 10^6^	[M − H]−	130.088	130.0874		
	*L. fermentum* FBR	2.48		3.24 × 10^5^	[M − H]−	130.088	130.0875		
	*L. reuteri* FBR	2.41		6.63 × 10^6^	[M − H]−	130.088	130.0875		
13	Raw BR	ND		ND	[M − H]−	ND	ND	C_5_H_10_N_2_O_3_	Glutamine
	*L. plantarum* FBR	ND		ND	[M − H]−	ND	ND		
	*L. fermentum* FBR	ND		ND	[M − H]−	ND	ND		
	*L. reuteri* FBR	1.10		1.62 × 10^4^	[M − H]−	145.063	145.0619		
14	Raw BR	ND		ND	[M − H]−	ND	ND	C_9_H_11_NO_3_	Tyrosine
	*L. plantarum* FBR	1.98		3.38 × 10^5^	[M − H]−	180.068	180.0667		
	*L. fermentum* FBR	ND		ND	[M − H]−	ND	ND		
	*L. reuteri* FBR	1.89		4.08 × 10^5^	[M − H]−	180.068	180.0667		
15	Raw BR	ND		ND	[M − H]−	ND	ND	C_4_H_9_NO_3_	Threonine
	*L. plantarum* FBR	ND		ND	[M − H]−	ND	ND		
	*L. fermentum* FBR	ND		ND	[M − H]−	ND	ND		
	*L. reuteri* FBR	1.14		1.14 × 10^5^	[M − H]−	118.052	118.051		
16	Raw BR	ND	ND		[M − H]−	ND	ND	C_4_H_8_N_2_O_3_	Asparagine
	*L. plantarum* FBR	1.10	6.8 × 10^4^		[M − H]−	131.047	131.0462		
	*L. fermentum* FBR	1.13	3.41 × 10^4^		[M − H]−	131.047	131.0464		
	*L. reuteri* FBR	1.13	3.01 × 10^5^		[M − H]−	131.047	131.0461		
17	Raw BR	ND	ND		[M − H]−	ND	ND	C_11_H_12_N_2_O_2_	Tryptophan
	*L. plantarum* FBR	7.64	6.19 × 10^5^		[M − H]−	203.084	203.0829		
	*L. fermentum* FBR	7.66	7.77 × 10^4^		[M − H]−	203.084	203.0832		
	*L. reuteri* FBR	7.61	2.78 × 10^6^		[M − H]−	203.084	203.0829		
18	Raw BR	ND	ND		[M + H]+	ND	ND	C_5_H_9_NO_2_	Proline
	*L. plantarum* FBR	ND	ND		[M + H]+	ND	ND		
	*L. fermentum* FBR	ND	ND		[M + H]+	ND	ND		
	*L. reuteri* FBR	0.86	7.90 × 10^5^		[M +H]+	116.07	116.0704		

ND—not detected, BR—brown rice, and FBR—fermented brown rice.

**Table 4:** Fatty acids detected in raw and LABs fermented brown rice.

S. No	Sample Name	Retention Time	Peak Area	Adduct/Charge	Precursor Mass	Found at Mass	Formula Finder Result	Fatty Acid
1	Raw BR	39.97	1.91 × 10^2^	[M − H]−	255.234	255.2331	C_16_H_32_O_2_	Palmitic Acid
	*L. plantarum* FBR	1.94	5.15 × 10^3^	[M − H]−	255.234	255.2331		
	*L. fermentum* FBR	28.03	4.28 × 10^3^	[M − H]−	255.234	255.2332		
	*L. reuteri* FBR	23.73	1.53 × 10^4^	[M − H]−	255.234	255.2332		
2	Raw BR	ND	ND	[M + H]+	ND	ND	C_5_H_10_O_2_	Valeric acid
	*L. plantarum* FBR	ND	ND	[M + H]+	ND	ND		
	*L. fermentum* FBR	ND	ND	[M + H]+	ND	ND		
	*L. reuteri* FBR	22.88	1.02 × 10^3^	[M + H]+	185.066	185.0663		
3	Raw BR	46.24	5.29 × 10^3^	[M − H]−	279.234	279.2332	C_18_H_32_O_2_	Linoleic Acid
	*L. plantarum* FBR	46.26	6.56 × 10^5^	[M − H]−	279.234	279.2332		
	*L. fermentum* FBR	46.24	6.16 × 10^5^	[M − H]−	279.234	279.2334		
	*L. reuteri* FBR	46.25	6.64 × 10^5^	[M − H]−	279.234	279.2334		
4	Raw BR	47.28	1.45 × 10^5^	[M + H]+	271.264	271.2637	C_17_H_34_O_2_	Heptadecanoic acid
	*L. plantarum* FBR	47.28	1.44 × 10^6^	[M + H]+	271.264	271.2636		
	*L. fermentum* FBR	47.26	1.44 × 10^6^	[M + H]+	271.264	271.2637		
	*L. reuteri* FBR	47.27	1.45 × 10^6^	[M + H]+	271.264	271.2638		
5	Raw BR	27.69	5.28 × 10^3^	[M − H]−	283.265	283.2644	C_18_H_36_O_2_	Stearic acid
	*L. plantarum* FBR	49.10	1.23 × 10^6^	[M − H]−	283.265	283.2645		
	*L. fermentum* FBR	49.09	1.20 × 10^6^	[M − H]−	283.265	283.2644		
	*L. reuteri* FBR	49.10	1.24 × 10^6^	[M − H]−	283.265	283.2645		
6	Raw BR	34.57	3.18 × 10^4^	[M − H]−	243.161	243.1605	C_13_H_24_O_4_	Tridecanedioic acid
	*L. plantarum* FBR	34.58	2.98 × 10^5^	[M − H]−	243.161	243.1606		
	*L. fermentum* FBR	34.58	2.90 × 10^5^	[M − H]−	243.161	243.1604		
	*L. reuteri* FBR	34.57	3.01 × 10^5^	[M − H]−	243.161	243.1605		
7	Raw BR	ND	ND	[M + H]+	ND	ND	C_12_H_20_O_3_	Traumatin
	*L. plantarum* FBR	32.80	2.64 × 10^5^	[M + H]+	213.149	213.1491		
	*L. fermentum* FBR	32.82	4.26 × 10^5^	[M + H]+	213.149	213.1492		
	*L. reuteri* FBR	32.82	5.20 × 10^5^	[M + H]+	213.149	213.1492		
8	Raw BR	ND	ND	[M − H]−	ND	ND	C_18_H_32_O_5_	Octadecadienoic acid/Corchorifatty acid F
	*L. plantarum* FBR	ND	ND	[M − H]−	ND	ND		
	*L. fermentum* FBR	ND	ND	[M − H]−	ND	ND		
	*L. reuteri* FBR	30.87	4.08 × 10^5^	[M − H]−	327.219	327.2181		
9	Raw BR	ND	ND	[M − H]−	ND	ND	C_6_H_12_O_4_	Mevalonic Acid
	*L. plantarum* FBR	3.49	7.39 × 10^4^	[M − H]−	147.067	147.0667		
	*L. fermentum* FBR	ND	ND	[M − H]−	ND	ND		
	*L. reuteri* FBR	3.47	3.13 × 10^5^	[M − H]−	147.067	147.0667		
10	Raw BR	22.59	3.99 × 10^4^	[M − H]−	187.099	187.0979	C_9_H_16_O_4_	Azelaic Acid
	*L. plantarum* FBR	22.50	2.77 × 10^5^	[M − H]−	187.099	187.0979		
	*L. fermentum* FBR	22.61	6.12 × 10^4^	[M − H]−	187.099	187.0978		
	*L. reuteri* FBR	22.50	6.25 × 10^5^	[M − H]−	187.099	187.0979		
11	Raw BR	ND	ND	[M − H]−	ND	ND	C_9_H_18_O_3_	9-Hydroxynonanoic acid
	*L. plantarum* FBR	23.49	3.10 × 10^4^	[M − H]−	173.119	173.1187		
	*L. fermentum* FBR	23.50	3.49 × 10^3^	[M − H]−	173.119	173.1188		
	*L. reuteri* FBR	23.48	1.39 × 10^5^	[M − H]−	173.119	173.1186		
12	Raw BR	39.06	2.45 × 10^3^	[M − H]−	313.24	313.2389	C_18_H_34_O_4_	Octadecanedioic acid
	*L. plantarum* FBR	39.07	3.52 × 10^5^	[M − H]−	313.24	313.2388		
	*L. fermentum* FBR	39.06	3.22 × 10^5^	[M − H]−	313.24	313.2389		
	*L. reuteri* FBR	39.06	3.83 × 10^5^	[M − H]−	313.24	313.2386		
13	Raw BR	ND	ND	[M − H]−	ND	ND	C_18_H_34_O_5_	Pinellic acid
	*L. plantarum* FBR	33.48	3.12 × 10^5^	[M − H]−	329.234	329.2337		
	*L. fermentum* FBR	33.48	3.54 × 10^4^	[M − H]−	329.234	329.2339		
	*L. reuteri* FBR	32.82	7.37 × 10^6^	[M − H]−	329.234	329.2331		

ND—not detected, BR—brown rice, and FBR—fermented brown rice.

## Data Availability

Data is contained within the article.

## Abbreviations

BR	Raw brown rice
FBR	Fermented brown rice
ROS	Reactive oxygen species
GABA	Gamma-aminobutyric acid

## References

[1] Poljsak B., Šuput D., Milisav I. (2013). Achieving the Balance between ROS and Antioxidants: When to Use the Synthetic Antioxidants. Oxidative Med. Cell. Longev..

[2] Tyagi A., Yeon S.-J., Daliri E., Chen X., Chelliah R., Oh D.-H. (2021). Untargeted Metabolomics of Korean Fermented Brown Rice Using UHPLC Q-TOF MS/MS Reveal an Abundance of Potential Dietary Antioxidative and Stress-Reducing Compounds. Antioxidants.

[3] Tyagi A., Daliri E.B.-M., Ofosu F.K., Yeon S.-J., Oh D.-H. (2020). Food-Derived Opioid Peptides in Human Health: A Review. Int. J. Mol. Sci..

[4] Sack M.N., Fyhrquist F.Y., Saijonmaa O.J., Fuster V., Kovacic J.C. (2017). Basic biology of oxidative stress and the cardiovascular system: Part 1 of a 3-part series. J. Am. Coll. Cardiol..

[5] Wang Y., Wu Y., Wang Y., Xu H., Mei X., Yu D., Wang Y., Li W. (2017). Antioxidant Properties of Probiotic Bacteria. Nutrients.

[6] Callcott E., Blanchard C., Oli P., Santhakumar A.B. (2018). Pigmented Rice-Derived Phenolic Compounds Reduce Biomarkers of Oxidative Stress and Inflammation in Human Umbilical Vein Endothelial Cells. Mol. Nutr. Food Res..

[7] Shabbir U., Arshad M., Sameen A., Oh D.-H. (2021). Crosstalk between Gut and Brain in Alzheimer’s Disease: The Role of Gut Microbiota Modulation Strategies. Nutrients.

[8] Okarter N., Liu R.H. (2010). Health Benefits of Whole Grain Phytochemicals. Crit. Rev. Food Sci. Nutr..

[9] Imam M.U., Musa S.N.A., Azmi N.H., Ismail M. (2012). Effects of White Rice, Brown Rice and Germinated Brown Rice on Antioxidant Status of Type 2 Diabetic Rats. Int. J. Mol. Sci..

[10] Pang Y., Ahmed S., Xu Y., Beta T., Zhu Z., Shao Y., Bao J. (2018). Bound phenolic compounds and antioxidant properties of whole grain and bran of white, red and black rice. Food Chem..

[11] Sandhu K.S., Punia S. (2017). Enhancement of bioactive compounds in barley cultivars by solid substrate fermentation. J. Food Meas. Charact..

[12] Salar R.K., Purewal S.S., Sandhu K.S. (2017). Fermented pearl millet (*Pennisetum glaucum*) with in vitro DNA damage protection activity, bioactive compounds and antioxidant potential. Food Res. Int..

[13] Salar R.K., Purewal S.S. (2016). Improvement of DNA damage protection and antioxidant activity of biotransformed pearl millet (Pennisetum glaucum) cultivar PUSA-415 using Aspergillus oryzae MTCC 3107. Biocatal. Agric. Biotechnol..

[14] Pradeep P., Sreerama Y.N. (2017). Soluble and bound phenolics of two different millet genera and their milled fractions: Comparative evaluation of antioxidant properties and inhibitory effects on starch hydrolysing enzyme activities. J. Funct. Foods.

[15] Chang X., Ye Y., Pan J., Lin Z., Qiu J., Guo X., Lu Y. (2018). Comparative assessment of phytochemical profiles and antioxidant activities in selected five varieties of wampee (*Clausena lansium*) fruits. Int. J. Food Sci. Technol..

[16] Xiang J., Zhang M., Apea-Bah F.B., Beta T. (2019). Hydroxycinnamic acid amide (HCAA) derivatives, flavonoid C-glycosides, phenolic acids and antioxidant properties of foxtail millet. Food Chem..

[17] Zeng Z., Hu X., McClements D.J., Luo S., Liu C., Gong E.S., Huang K. (2019). Hydrothermal stability of phenolic extracts of brown rice. Food Chem..

[18] Daliri E.B.-M., Ofosu F.K., Chelliah R., Kim J.-H., Kim J.-R., Yoo D., Oh D.-H. (2020). Untargeted Metabolomics of Fermented Rice Using UHPLC Q-TOF MS/MS Reveals an Abundance of Potential Antihypertensive Compounds. Foods.

[19] Ti H., Guo J., Zhang R., Wei Z., Liu L., Bai Y., Zhang M. (2015). Phenolic profiles and antioxidant activity in four tissue fractions of whole brown rice. RSC Adv..

[20] Metsalu T., Vilo J. (2015). ClustVis: A web tool for visualizing clustering of multivariate data using Principal Component Analysis and heatmap. Nucleic Acids Res..

[21] Ilowefah M., Bakar J., Ghazali H.M., Muhammad K. (2017). Enhancement of Nutritional and Antioxidant Properties of Brown Rice Flour Through Solid-State Yeast Fermentation. Cereal Chem. J..

[22] Gong E.S., Liu C., Li B., Zhou W., Chen H., Li T., Wu J., Zeng Z., Wang Y., Si X. (2020). Phytochemical profiles of rice and their cellular antioxidant activity against ABAP induced oxidative stress in human hepatocellular carcinoma HepG2 cells. Food Chem..

[23] Huang S.-H., Ng L.-T. (2012). Quantification of polyphenolic content and bioactive constituents of some commercial rice varieties in Taiwan. J. Food Compos. Anal..

[24] Gong E.S., Luo S.J., Li T., Liu C.M., Zhang G.W., Chen J., Zeng Z.C., Liu R.H. (2017). Phytochemical profiles and antioxidant activity of brown rice varieties. Food Chem..

[25] Ghasemzadeh A., Jaafar H.Z.E., Juraimi A.S., Tayebi-Meigooni A. (2015). Comparative Evaluation of Different Extraction Techniques and Solvents for the Assay of Phytochemicals and Antioxidant Activity of Hashemi Rice Bran. Molecules.

[26] Lin C.-C. (2019). Antioxidant properties and antibacterial activity of fermented Monascus purpureus extracts. Food Biosci..

[27] Rao S., Santhakumar A.B., Chinkwo K., Snell P., Oli P., Blanchard C. (2020). Rice phenolic compounds and their response to variability in growing conditions. Cereal Chem. J..

[28] Dai T., Chen J., McClements D.J., Hu P., Ye X., Liu C., Li T. (2019). Protein–polyphenol interactions enhance the antioxidant capacity of phenolics: Analysis of rice glutelin–procyanidin dimer interactions. Food Funct..

[29] Janarny G., Gunathilake K. (2020). Changes in rice bran bioactives, their bioactivity, bioaccessibility and bioavailability with solid-state fermentation by Rhizopus oryzae. Biocatal. Agric. Biotechnol..

[30] Zeki E., KÖSE S.B.E., GARLI S., ŞAHİNDOKUYUCU F.K. (2021). The Antioxidant Effect of p-Coumaric Acid Against Toluene-Induced Oxidative Stress in Rats. Kahramanmaraş Sütçü İmam Üniversitesi Tarım ve Doğa Dergisi.

[31] El-Beltagi H.S., Mohamed H.I., Sofy M.R. (2020). Role of Ascorbic acid, Glutathione and Proline Applied as Singly or in Sequence Combination in Improving Chickpea Plant through Physiological Change and Antioxidant Defense under Different Levels of Irrigation Intervals. Molecules.

[32] Anlar H.G., Preedy V.R. (2020). Chapter 23-Cinnamic Acid as a Dietary Antioxidant in Diabetes Treatment.

[33] Ahmadi N., Safari S., Mirazi N., Karimi S.A., Komaki A. (2021). Effects of vanillic acid on Aβ1-40-induced oxidative stress and learning and memory deficit in male rats. Brain Res. Bull..

[34] Adebo O.A., Njobeh P.B., Adebiyi J.A., Gbashi S., Phoku J.Z., Kayitesi E. (2017). Fermented Pulse-Based Food Products in Developing Nations as Functional Foods and Ingredients. Functional Food-Improve Health through Adequate Food.

[35] Gao Y., Guo X., Liu Y., Zhang M., Zhang R., Abbasi A.M., You L., Li T., Liu R.H. (2018). Comparative assessment of phytochemical profile, antioxidant capacity and anti-proliferative activity in different varieties of brown rice (Oryza sativa L.). LWT.

[36] Girard-Lalancette K., Pichette A., Legault J. (2009). Sensitive cell-based assay using DCFH oxidation for the determination of pro- and antioxidant properties of compounds and mixtures: Analysis of fruit and vegetable juices. Food Chem..

[37] Chen P.X., Zhang H., Marcone M.F., Pauls K.P., Liu R., Tang Y., Zhang B., Renaud J.B., Tsao R. (2017). Anti-inflammatory effects of phenolic-rich cranberry bean ( Phaseolus vulgaris L.) extracts and enhanced cellular antioxidant enzyme activities in Caco-2 cells. J. Funct. Foods.

[38] Grauzdytė D., Pukalskas A., Viranaicken W., El Kalamouni C., Venskutonis P.R. (2018). Protective effects of Phyllanthus phillyreifolius extracts against hydrogen peroxide induced oxidative stress in HEK293 cells. PLoS ONE.

[39] Kellett M.E., Greenspan P., Pegg R.B. (2018). Modification of the cellular antioxidant activity (CAA) assay to study phenolic antioxidants in a Caco-2 cell line. Food Chem..

